# The Spanish Intergenerational Study: Beliefs, Stereotypes, and Metacognition about Older People and Grandparents to Tackle Ageism

**DOI:** 10.3390/geriatrics6030087

**Published:** 2021-09-03

**Authors:** Aida Muntsant, Paula Ramírez-Boix, Rocío Leal-Campanario, Francisco Javier Alcaín, Lydia Giménez-Llort

**Affiliations:** 1 Department of Psychiatry and Forensic Medicine, School of Medicine, Autonomous University of Barcelona, E-080193 Barcelona, Spain; aida.muntsant@uab.cat (A.M.); paulaboix@gmail.com (P.R.-B.); 2 Institut de Neurociències, Autonomous University of Barcelona, E-080193 Barcelona, Spain; 3 División de Neurociencias, Universidad Pablo de Olavide, 41013 Sevilla, Spain; rleacam@upo.es; 4 Department of Medical Sciences, Faculty of Medicine, University of Castilla-La Mancha, 13071 Ciudad Real, Spain; franciscoj.alcain@uclm.es; 5 Oxidative Stress and Neurodegeneration Group, Regional Centre for Biomedical Research CRIB, University of Castilla-La Mancha, 13071 Ciudad Real, Spain

**Keywords:** ageism, stereotypes, discrimination, old population, grandparents, strategies, decade of healthy ageing

## Abstract

Ageism can be seen as systematic stereotypes, prejudice, and discrimination of people because of their age. For a long time, society has accepted negative stereotypes as a norm. When referring to older adults, the United Nations Global Report on Ageism warns about a severe impact. The Intergenerational Study for a Healthy Aging, a questionnaire about believes, stereotypes, and knowledge about older people and grandparents, was administered to 326 Spanish biology and medical students. Here we report the results of stereotype analysis through adjective qualification of the youth and older people performed before the survey. Content analysis of two open questions about metacognition at the end of the survey is also presented. The results show that: (1) The questionnaire promoted metacognition; (2) Positive metacognition toward grandparents was higher than for the general old population; (3) Most participants were not conscious about ageism; (4) Gender was a key factor—male students were more ageist than females; (5) The feeling of guilt was higher in the questionnaire about older people; (6) The metacognition exercise elicited thoughts and, in few cases, the need to take action to tackle ageism. In conclusion, both activities promoted active thoughts about older people vs. grandparents and helped participants realize unconscious ageism—specifically toward the older population—serving as an awareness activity that may help tackle ageism.

## 1. Introduction

The term ageism was first used in 1969 to describe negative attitudes, prejudice, and discrimination toward older people [1]. However, different authors have defined it over time and nowadays also include those considered too young for being or participating in something. In the context of the Decade of Healthy Aging (2021–2030) [2], the United Nations (UN) Global Report on Ageism warned about the severity of its impact on the older population [3]. Ageism is a personal revulsion to and distaste for growing old, disease, disability, and fear of powerlessness, uselessness, and death. It can be seen as a process of systematic stereotyping and discrimination against people because they are old and one-self confronting the aging process. It also allows younger generations to see older people as different from themselves [4]. It can result in one group controlling another group by eliminating their liberties and opportunities [5]. Generally, individuals perceived as old automatically are categorized as senile, antiquated, and obstinate [6]. These facts define ageism analogously to racism and sexism because all three employ stereotypes to denigrate members of a group [1,7]. However, ageism is different from the other two -isms because everyone may become an older person if they live long enough. It has not been studied so widely because it is a relatively new and also a subtle concept. In fact, some studies pointed out that ageism is possibly more prevalent than sexism and racism [8]. Moreover, the general population, including most scientists, has accepted this negative stereotype as a norm for a long time. Researchers know much about the other prejudiced stereotypes, but there is little information about stereotyping based on age. For example, terms such as ‘elderly people’ are considered outdated, disrespectful, or offensive, but lack of awareness makes it still common in use, and few people consider exchanging the word or phrase for ‘older adults’ or ‘older people’ [9].

Spain is one of the leading countries with the oldest population, which is in constant growth. In 2020, adults in Spain over 65 accounted for 19.58% of the population compared to 17.90% in 2013 and 11.24% in 1981 [10]. As more countries experience the novelty of population aging, with an increasing percentage of older people, ageism has gradually become an important issue for scholars and public policymakers. Despite the change in demographics, the studies still indicate cultural stereotypes around old age (the people) and aging (the process) that express a negative view [11], pointing out that these stereotypes and prejudices by professionals and families result in an increased risk of discrimination and social exclusion of older people. Notwithstanding the relevance of grandparents–grandchildren bonds, college students have been reported to be the group who presented the most prevalent and negative ageist attitudes [12,13].

In recent years several strategies to reduce ageism have been studied. Interventions involving education and intergenerational contact have encouraged a significant reduction in ageism attitudes [14,15,16,17]. Regarding health care, older people are the ones who consume more health care resources in the so-called ‘silver economy’. For this reason, factors associated with health students’ and health care providers’ positive attitudes toward the older people’s health situation have been reviewed [18]. Interventions to increase consciousness about ageism have been also reported in health care professionals and students [19]. For example, interaction with real patients and empathy skills seem to be good interventions associated with positive attitude change in medical [20] and nursing [21] students toward older adults. These kinds of intervention have been also been proven to be useful regarding health care professionals’ attitudes [22,23].

On the other hand, the concept and term metacognition, which emerged as an object of studies in psychology in the late 1970s from Flavell’s research on cognitive processes [24,25], may play a role in tackling and fighting ageism. The concept refers to the cognitive processes involved in thinking about what we think, and it is characterized by a high level of awareness and voluntary control. Information about cognition itself implies that a person can understand the results of an activity. According to Flavell, metacognition exists because the human thinks. For this reason, someone is susceptible to make mistakes and needs some mechanism that allows for adjusting these errors. Additionally, metacognition is necessary for people because it will enable them to plan and make decisions in their life [24,25].

The new rationale of our intergenerational study is based on our hypothesis that given a group of grandparents and grandchildren, they would receive better mutual evaluations when matched according to their family bonds than if the same participants were unmatched. In the present report, we contribute to the literature asking students to think about their stereotypes; the answers they wrote about their knowledge/beliefs about older people and those about their grandparents may help university students to become more aware of ageist attitudes, feel closer to older adults and help tackle ageism. Our aim is to provide new evidence that intergenerational studies assessing older people, in general, and those with family bonds, would help young participants to become aware of ageist thoughts and attitudes, mainly toward the general older population (macrosystem) with whom they are not related personally (microsystem) with bonds.

## 2. Materials and Methods

### 2.1. The Spanish Intergenerational Study

Since 2008, ‘The Spanish Intergenerational Study’ on the grandparents–grandchildren bond has used Bronfenbrenner’s ecological approach to find the clues for positive family relationships at the microsystem level that, when translated into macrosystem scenarios, may help minimize the ageism toward the older population. Students at several Spanish universities enrolled in this study that has also been performed in universities of other countries. The preliminary results revealed that the Spanish Intergenerational Study questionnaire induced young people to conduct a more reflexive and auto-critical analysis of their intergenerational relationships than those shown toward other unrelated older people, which positively challenged ageism [26]. For all these reasons, this study’s first aim was to understand better what young people think about the older population and if male students were more ageist than females. First, participants were asked to answer two questionnaires about beliefs and knowledge about older people and their grandparents. Then, the task ended with an exercise of metacognition where participants answered two open questions.

### 2.2. Data Collection

The present report is a retrospective analysis of a specific part of the results of the Spanish Intergenerational Study. The sample and data used in the present report correspond to selecting a homogenous and convenience sample of 326 biology (165) and medical (161) second and third-year students from two neighboring south Spanish universities that were assessed in person, using a paper-pencil survey, in 2010. Of the participants, 40.8% were male and 59.2% were female. The average age of respondents was 21.02 ± 0.12 years old (min 19, max 38).

The data collection was conducted in Spanish and then translated to English for the manuscript writing. Before the survey, participants received both oral and written information about the Spanish Intergenerational Study. The students that volunteered to participate signed the informed consent, assuring them that their participation was voluntary and anonymous, that they could withdraw from participation at any time, and that their response would be kept confidential.

### 2.3. Stereotypes Analysis through Adjective Qualification of the Youth and Older People

In a first exercise, students’ stereotypes about youth and older people were investigated through adjective qualification of the youth and older people. Students were asked to provide a quick answer, in 30 s, of 4 adjectives describing what ‘Youth’ and ‘Older people’ meant to them. For the analysis, a literal transcript of the answers was conducted, then the number of adjectives was counted, and two independent observers evaluated their positive/negative value. In the case of inter-rater disagreement, the ‘Diccionario de la lengua española’ (Dictionary of the Spanish language) of the RAE, Real Academia Española (Royal Spanish Academy, Madrid, Spain), or a third independent observer were consulted.

### 2.4. Surveys: The Spanish Intergenerational Study on Older People and Grandparents

After that, students answered the ‘Intergenerational Study’ consisting of two questionnaires about beliefs and knowledge about older adults elaborated by our research group based on standard questionnaires about older people and intergenerational relationships of IMSERSO (Instituto de Mayores y Servicios Sociales, Spanish Institute for the older people and social services). The first questionnaire was about older people in general, and the other one was about their grandparents. At the end of the questionnaires, the participants answered two open questions, as described below. In the present work, we report the analysis of the self-report to these answers.

For the older people questionnaire:

(1) Summarize, in one sentence, what you think about older people.
(2) Explain, briefly, how do you feel after answering this questionnaire.

For the grandparents’ questionnaire:

(1) Summarize, in one sentence, what you think about your grandfather/grandmother.
(2) Explain, briefly, how do you feel after answering this questionnaire.

**Figure 1:**
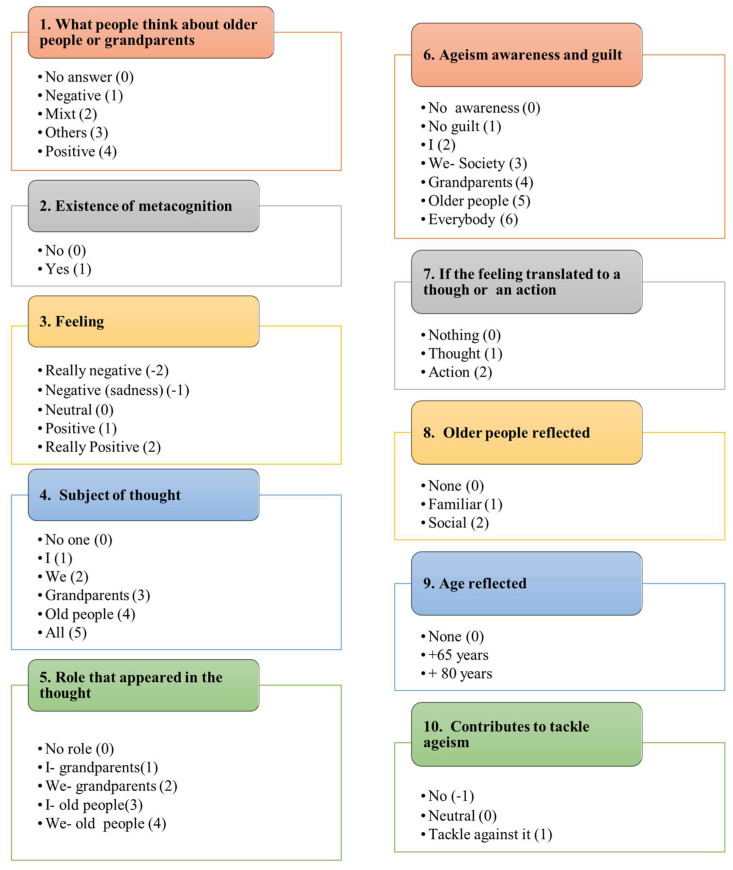
Content analysis to classify the participants’ answers to the open questions referring to the two questionnaires (older people and grandparents) of the Intergenerational Survey. Codes/items 1–6 and 8–10 were pre-established (1–6, 8–10) and code/item 7 was emergent (7).

For the content analysis, first, a literal transcript of the answers was conducted. A thorough reading of the transcripts was carried out to identify the fragments of analytical interest; all the corresponding fragments were grouped with the same code; finally, the information obtained was analyzed and interpreted [27]. A book of codes was established (see Figure 1) with those previously established (questions/codes 1–6 and 8–10) and the one that emerged during the analysis (question/code 7).

1. What the participants thought about older people or their grandparents (depending on the questionnaire analyzed).

First, students’ thoughts about older people and their grandparents were scored with an age stereotype negativity scale: 0, no answer; 1, a negative question; 2, mixed of answers (negative, positive, sadness); 3, other answers (neutrals); 4, positive answers.

2. The existence of metacognition.

The existence of metacognition in the answer, defined as ‘thinking about what they thought’, was scored as presence/absence: 0, lack of metacognition; 1, the existence of metacognition.

3. The feelings elicited after answering the questionnaires.

The feelings elicited were scored as follows: −2, very negative feelings; −1, negative feelings (sadness); 0, neutral feelings; 1, positive feelings; 2, very positive feelings.

4. The subject of the thought.

The subject of the thought when people answer the question was scored as 0; if there was no subject; 1, if the subject was ‘I’; 2, if the subjects were ‘We’; 3, if the subjects were grandparents; 4, if the subject were ‘older people’; 5, if the subject was ‘everyone’.

5. The role that appeared in the thought.

The role that appeared in the thought was also analyzed: 0, no role; 1, the family role such as I—grandparent; 2, the family role such as we—grandparents; 3, the social role such as I—older people; 4, the social role such as we—older people.

6. If there was ageism awareness and who was considered guilty of this ageism.

The sixth item studied if there was ageism awareness and who was guilty of this ageism. No awareness was coded as 0; the respondents who considered themselves not guilty were coded as 1; respondents who considered themselves guilty were coded as 2; respondents who considered society in general as guilty were coded as 3; people who thought that the guilty people were their grandparents were coded as 4; respondents who thought that the guilty people were older people, in general, were coded as 5; finally, people who thought that everyone is guilty, were coded as 6.

7. If the feeling was translated to a thought or an action.

Another item, which emerged during the content analysis, was to observe if the feeling that people had when answering the questionnaire and thinking about it was translated into a thought or an action. If it was not translated into anything, it was coded as 0; if the feelings were translated into thought, it was coded as 1; and finally, if the feelings were translated into action, it was coded as 2.

8. What kind of older person was reflected in the answer?

Another item was related to the kind of older people reflected in the answer. If the answer did not reflect any kind of older people, it was coded as 0; if the answer reflected a familiar older person, it was coded as 1; and if the answer reflected a social older person, it was coded as 2.

9. What age of older people was reflected in the answer?

The kind of older people reflected was evaluated with the following answers. If there was no age reflected, the answer was coded as 0; if the answer reflected older people in the 3rd and 4th age (+65 years), it was coded as 1; if the answer reflected older people in the 4th age (+80 years), it was coded as 2.

10. If the answer to the questionnaires could help to tackle ageism.

Finally, whether answering the questionnaires and completing the exercise of metacognition helped to tackle ageism was scored as follows: if completing the exercise worsened ageism, it was coded as −1; if answering the questionnaire neither worsened nor helped to tackle ageism, it was coded as 0; and if answering the questions helped to tackle ageism, it was coded as 1.

All these items were taken into account two times, one for the answers after the questionnaire of older people and the other after the questionnaire of grandparents.

### 2.5. Statistics

Results are expressed as mean ± S.E.M or as incidence. SPSS 15.0 software was used. Two independent measures between men and women were compared by Student’s *t*-test, while comparisons for related answers were made with the paired *t*-test. The chi-Square and marginal homogeneity model tests assessed differences in the no parametric responses, respectively. *p* < 0.05 was considered statistically significant.

## 3. Results

### 3.1. Socio-Demography

Basic demographic background information, including the city of birth, age, and gender, was collected. Only the students born in Cordoba (students of biology) and Seville (students of medicine) were considered.

### 3.2. Stereotype Analysis through Adding Adjectives to the Youth and Older People

**Figure 2:**
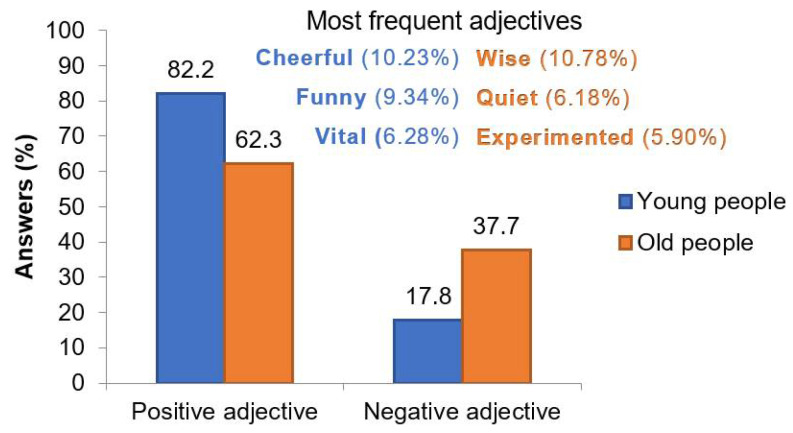
Stereotype analysis through identifying the youth and the older people with adjectives. The bars illustrate the percentage of positive and negative adjectives for young and older people. The most frequent adjectives are included as an inset, and their percentages are indicated.

When participants were asked to think about four adjectives for young people and four adjectives for older people, not everybody found the four adjectives. In general, people provided more adjectives about young people (3.43 ± 0.063) than older people (3.35 ± 0.067) (*t*-test, *p* = 0.021). However, the results indicated that these differences were because of the male’s answers, as gender differences in the number of adjectives about older people were noticed (*t*-test, *p* = 0.016). Males answered 3.32 ± 0.107 adjectives for young people and 3.14 ± 0.117 adjectives for older people in comparison with women, who answered 3.51 ± 0.76 for young people and 3.49 ± 0.079 for older people. A total of 82.2% of the adjectives written for young people were positive compared to 62.3% for older people (Figure 2).

An eight-scale was used to quantify the total number of adjectives and then transformed to a percentage. A figure of 0 represented that all the adjectives were negative, and 100 that all adjectives were positive. In this study, young people obtained more positive punctuation (77.6 ± 1.21) than older people, who received 60.3 ± 1.64 (Student *t*-test, *p* = 0.01). On the other hand, although females wrote fewer negative stereotypes than males for older people, no significant gender differences were detected. Additionally, no statistical differences between the students’ answers of both universities were observed.

The three most repeated adjectives from students for young and older people were positives. They described young people as cheerful (10.23%), funny (9.34%), and vital (6.28%). On the other side, students described older people as wise (10.78%), quiet (6.18%), and experimented (5.90%).

### 3.3. Survey: The Spanish Intergenerational Study—Older People Questionnaire

#### 3.3.1. What Do Students Think about Older People?

**Figure 3:**
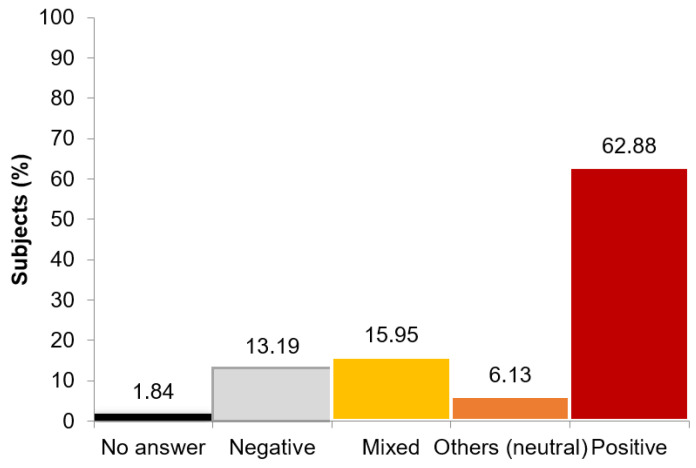
The Spanish Intergenerational Study—Older people questionnaire: Percentage of subjects providing a positive, neutral, mixed, negative, or no answer on the open questions of the older people questionnaire.

When we analyzed the first question to evaluate the level of aging with the aging stereotype scale, we observed that all the answers were related to aging (the process) more than to older people (the subjects). Therefore, for this reason, we decided to measure the stereotypes that students self-reported about aging (Figure 3). In general, medical and biology students perceived aging in a positive way (62.88%). However, in almost 30% of the answers, some negative connotations appeared (negative thinking or mixed negative and positive thinking).

Some examples of answers about aging:

- Negative stereotypes: “No se tienen en cuenta por la sociedad, están infravalorados.” “They are not taken into account by the society; they are underestimated.”
- Mixed stereotypes: “Son gente sabia pero débil, necesitan el cariño de su familia.” “They are wise people but also weak; they need the love of their families.”
- Other stereotypes (neutral): “Personas normales e iguales a los demás.” “Normal people and equal to others.”
- Positive stereotypes: “Personas que pueden transmitir conocimiento basado en sus experiencias y enriquecernos.” “People who can impart knowledge base don their experiences and enrich us.”

Results from the ten different items taken into account to analyze the open answers of both questionnaires were presented in Table 1.

#### 3.3.2. The Existence of Metacognition

After answering the questionnaire about older people, most participants (93.3%) performed the metacognition exercise. This fact indicates that asking participants to think about their thoughts and feelings during the questionnaire successfully encouraged people to test and report their metacognition.

#### 3.3.3. What Feelings Arise When Answering the Questionnaires?

In most cases, answering the questionnaire produced a neutral (40.8%) or a positive feeling (37.8%). Other answers represented a negative feeling (13.5%), and a few referred to a sad feeling (6.6%).

#### 3.3.4. Who Is the Subject of the Thought?

In most cases, the participants who answered the questions were the subject of the thought (97%).

#### 3.3.5. What Role Appeared in the Thought?

In most answers, any role was detected. In those where a role was expressed, the percentage was similar between I–grandparents (17.4%) and older people (11.5%). It is interesting to note that grandparents appeared in the metacognition exercise even though the questionnaire was about older people in general. In this case, we observed gender differences, since 71.2% of males’ answers did not refer to any role, compared to 56.4% of females (χ^2^, *p* = 0.05).

#### 3.3.6. Is There Ageism Awareness? Who Is Guilty of This Ageism?

The majority of answers indicate that participants were not conscious of ageism (83.9%). In the answers where ageism awareness is referred to, students that blame themselves are in the same ratio as those who blame society (7.2% and 6.6%, respectively). Finally, although the number was meager (0.3%), some people blame older people and explain that ageism exists because some older people exhibit poor behavior.

#### 3.3.7. Is the Feeling Translated into a Thought or an Action?

On the other hand, when we measured if the feeling was translated into a thought or an action, the feeling was translated into thought in around half the people asked (45.4%). In very few cases, completing the exercise of metacognition led to some people developing the intention to carry out some action to tackle ageism (2%).

Moreover, we observed gender differences in this item. In this case, the feeling was translated into thought in 35.2% of men and 52.5% of women (χ^2,^ *p* = 0.009).

#### 3.3.8. What Kinds of Older People Are Reflected in the Answer?

Although several answers did not reflect any kind of older person (61.5%), the types of older people represented were more or less the same between the familiar and social older people (17.4% and 21.1%, respectively). In this case, it was the same as in the role item: grandparents (familiar older people) were present in students’ thoughts, although the questionnaire was about older adults in general. Moreover, we also observed differences in the answers between males and women (χ^2^, *p* = 0.012). A total of 71.2% of males’ answers did not reflect older people compared to 54.7% of women’s answers.

#### 3.3.9. What Age Is Reflected in the Answer?

More than half of the answers did not reflect any age (61.5%), but when they did, most cases talked about people older than 65 years in general (36.9%). We also observed gender differences, where males did not reflect any age in the answer in 71.2% of cases compared to 54.7% of women (χ^2^, *p* = 0.013).

#### 3.3.10. Could Answering the Questionnaires Help to Tackle Ageism?

Finally, one of the aims of this study was to investigate whether carrying out metacognition could help tackle ageism. We observed that completing the exercise helped tackle ageism in a significant number of students asked (63.5%) and did not worsen it in any of the cases.

Moreover, we have also observed gender differences in the answers to the older people’s questionnaire (χ^2^, *p* = 0.01). In this case, despite the fact that the participants were requested to answer the open questions with a brief sentence, the metacognition seemed to elicit more thoughts, feelings, and attitudes to tackle ageism in women than men (52.8% in males vs. 70.9% in females).

### 3.4. Survey: Spanish Intergenerational Study—Grandparents’ Questionnaire

#### 3.4.1. What Do Students Think about Their Grandparents?

**Figure 4:**
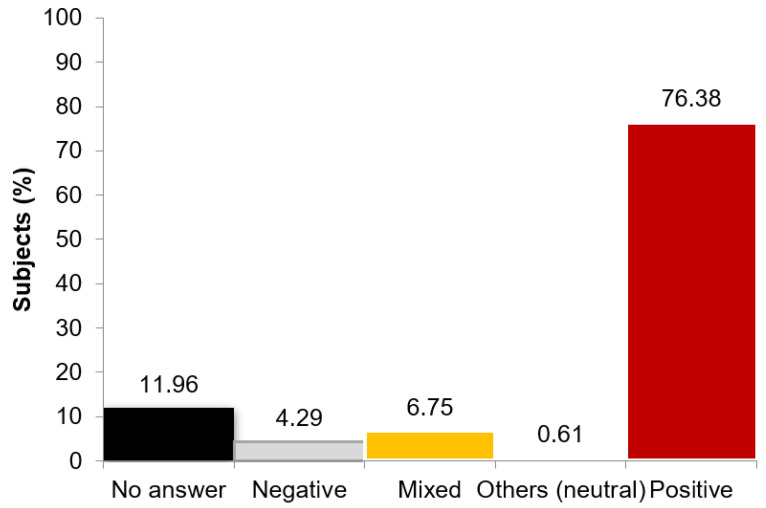
The Spanish Intergenerational Study–Grandparents’ questionnaire: Percentage of subjects providing a positive, neutral, mixed, negative or no answer to the open questions of the grandparents’ questionnaire.

When we analyzed the first question to evaluate the level of aging with the age stereotype negativity scale, we observed that, in general, medical and biology students have a favorable view of their grandparents (Figure 4). Only about 10% of the answers contain negative connotations (negative thinking or negative and positive thinking).

Examples of answers:

1. Negative stereotypes: ‘Terco, mulo, cascarrabias y posesivo’. Stubborn, mule, moody and possessive.
2. Mixed stereotypes: ‘Es una persona cabezota y autoritaria pero es buena persona y siempre se ha portado muy bien conmigo’. It is a stubborn and authoritarian person, but he is a good person and has always been very nice to me.
3. Other stereotypes (neutral): ‘Es un miembro más de la familia igual como cualquier otro.’ It is a member of the family just like any other.
4. Positive stereotypes: ‘La mejor del mundo’. The best of the world.

#### 3.4.2. The Existence of Metacognition

Most people performed the metacognition exercise after answering the second part of the survey corresponding to the grandparents’ questionnaire. This fact indicates that answering the questionnaire and requesting participants to think about their feelings encouraged them to think about older adults (84.1%). A total of 15.9% of students did not perform the exercise, a fact that could be explained as probably due to the exercise being at the end of the questionnaire, and some students could have been tired.

#### 3.4.3. What Feelings Arise When Answering the Questionnaires?

More answers represented a sad feeling than a negative feeling (24.5% and 6.2%, respectively). However, half of the students had a positive or a very positive feeling after answering the questionnaire.

#### 3.4.4. Who Is the Subject of the Thought?

As before, the participants who answered this question were the subject of the thought (96.7%).

#### 3.4.5. What Role Appeared in the Thought?

In more than half of the answers (56.2%), any role could be detected. The role ‘I–grandparents’ (40.9%) was the highest reported in contrast to the one involving older adults, scarcely mentioned.

#### 3.4.6. Is There Ageism Awareness? Who Is Guilty of This Ageism?

Although almost all the answers indicate that participants were not conscious of ageism (91.6%), in those answers referring to ageism awareness, students blame themselves more than society (4.7% and 2.9%, respectively).

#### 3.4.7. Is the Feeling Translated into a Thought or an Action?

On the other hand, when we measured if the feeling was translated into a thought or an action, the feeling was translated into thought in about half the participants asked (44.5%). Despite representing very few cases, the exercise of metacognition also induced the intention to take action to tackle ageism (2.6%). Moreover, we observed gender differences in this item. Thus, the feeling was translated into thought in 38.9% of men and 48.4% of women (χ^2,^ *p* = 0.013).

#### 3.4.8. What Kind of Older Person Is Reflected in the Answer?

Although half of the answers did not reflect any (57.7%), in those who did so, the kind of older person that was mentioned was the familiar one (grandparents) (38.3%). However, older people in general (social) were almost not present in the answers.

#### 3.4.9. What Age Is Reflected in the Answer?

More than half of the answers did not reflect any age, but most cases talked about people older than 65 years when they did. Only some cases spoke about older people of more than 80 years.

#### 3.4.10. Answering the Questionnaires Could Help to Tackle Ageism?

Finally, one of the aims of this study was to know if practicing metacognition could help tackle ageism. In most cases, it did so.

### 3.5. Comparison of Older People with Grandparents

There are interesting results regarding differences in the way students think about older people in general and their grandparents in particular (Table 1). Thus, although grandparents are also older people, there is a family bond.

First, differences were observed in the stereotypes for grandparents (marginal homogeneity test, *p* = 0.046), since more positive stereotypes were used when students thought about their grandparents.

Most people performed the exercise of metacognition after answering the test. However, we observed a reduction in the number of people that performed the exercise when they were asked about grandparents (marginal homogeneity test, *p* = 0.00). As mentioned, it could be explained because the questions about grandparents were at the end of the exercise, and some students might be too tired to fill in the open questions or openly express their opinions.

Differences in the feelings raised after the two questionnaires, for older people and grandparents, were also observed (marginal homogeneity test, *p* = 0.008). There were more answers about negative feelings in the questionnaire about older people, whereas sadness was more elicited after the one about grandparents. After answering the questionnaire on older people, the reports also obtained a significant number of neutral questions; however, positive and very positive answers were more representative of the grandparents’ questionnaire.

Other differences referred to the different roles expressed in the thought (marginal homogeneity test, *p* = 0.000). Although most answers did not detect any role, when they did so, the percentage was similar between ‘I–grandparents’ and ‘I–older people’ when participants were asked about older people. However, the role ‘I–grandparents’ was seen more when participants had been asked about grandparents.

On the other hand, although most answers about older people and grandparents indicate that participants were not conscious of ageism, there were more answers about ageism consciousness when we asked about older people. Moreover, those blaming themselves were in the same ratio as those blaming society. When we asked about grandparents, the number of conscious people about ageism was lower. Here, participants blamed themselves more than society (marginal homogeneity test, *p* = 0.001).

Several answers did not refer to older people. However, when we studied those mentioned, they equally referred to familiar and social older people. However, after the questionnaire about grandparents, most answers were only related to familiar older people (marginal homogeneity test *p* = 0.03).

Finally, we observed that in both cases, completing the exercise of metacognition helped to tackle ageism. However, the number was higher in grandparents (marginal homogeneity test *p* = 0.000).

## 4. Discussion

Prior studies have shown that the general population has an ambivalent view toward older adults. Thus, they describe them with positive features such as warm and kind, but, at the same time, negative stereotypes such as incompetent or incapable are also observed [28]. College students have been reported to present the most prevalent and negative ageist attitudes [12,13]. The authors explained this by various stereotyped images of old age acquired from media and the lack of interactions with older adults. Some meta-analytic studies have also indicated that younger people possess more ageist attitudes than older ones [29,30]. These cultural stereotypes around aging have also been observed in Spain [31], and attitudes toward older adults among students of health care-related studies at the University of Salamanca have been analyzed [32]. In the present ‘Spanish Intergenerational Study’, despite older people being described with less positive adjectives than young people, the medicine and biology students presented a rather positive attitude since the three most repeated adjectives for older people were positive. In general, students thought that older people are wise, quiet, and experimented. Although we did not use the typical questionnaire to evaluate the ageism stereotypes, our results agree with those from other authors who noticed that health-related students presented a low percentage of negative stereotypes of older adults [33,34]. In fact, more contact with older adults has been correlated with a more positive perception of aging [35]. This has been explained because ageism often originates from a lack of understanding about the aging process and older adults [12,13,36,37]. In this respect, as shown by the survey, the participants indicated that their relationship with grandparents was relevant to their lives. On the other hand, the formative academic process related to health care and biological sciences may also be critical concerning the way the students see the general population, as also indicated by other authors from different countries [38,39,40,41,42]. It will be interesting to analyze the responses to our survey in students from other geographical areas in Spain and other countries where the study has been implemented, and also to explore in the humanities fields, to see if the results are the same, or if they change according to the area/field of study.

The second objective of this study was to understand better how medical students see older people, how they feel about them, and the role of family bonds in these aspects when the questions refer to their grandparents. To the best of our knowledge, the Spanish Intergenerational Study is the first to address these questions about both aging populations and grandparents at the same time. Therefore, two questionnaires about beliefs and knowledge about older people and their grandparents were administered. The survey also included a metacognition exercise as the last closing task, using two open questions. According to our hypothesis on the positive role of intergenerational bonds, the results showed that grandparents were better perceived than older people. For instance, more negative age stereotypes were referred to when participants were asked about older people than their grandparents. Moreover, the questionnaires confirmed that participants considered their grandparents as very important in their life. Although participants were asked about older people in general in the first questionnaire, the familiar role was as important as a social role in the students’ answers. In general, grandparents were present in a great number of answers about older people. In contrast, when the second questionnaire asked about grandparents, almost all the questions were related to students’ grandparents, and older people were hardly referred. One reason that could explain this fact is that a higher level of interaction with older people could result in more positive attitudes toward them. As some studies explained, more interaction suggests a more positive attitude toward older people [35]. This will agree with Kite and Johnson [43], who reported that when specific information about a target person was provided, compared with a general target such as an older person, age-based attitude differences were significantly reduced.

On the other hand, we noticed that in almost all the answers, females (representing 71.4% of the students registered for health degrees during the 2020–2021 course [44]) presented less ageist attitudes than males when we asked about older people in general. Males showed more negative stereotypes than women or they presented more neutral answers. This would agree with several studies that observed significant differences between views that men and women have of the older population, with females being less ageist than males [36,37,45]. Interestingly, in the present work, when we asked about grandparents, this gender difference was not so noticeable, and gender differences were only raised in the open questions. There, female participants self-reported that answering the survey was translated into a thought or the need to take a positive action toward their grandparents. This supports the relevance of the role of gender not only in thoughts, feelings, and attitudes but also in the extent to which people feel open to express their metacognitive process and their outcomes. Here, it would be important to note that gender differences in empathy and moral dilemmas may explain the results, and conversely, unveil certain gender biases. Thus, according to recent work by Baez et al. [46], despite experimental studies and neuropsychological assessments being inconsistent regarding gender effects—probably due to separate and small sample populations—their two large-scale studies on self-reported data consistently indicate greater empathy in females [46]. In their work, the self-reports of females portrayed themselves as more empathic, and utilitarian responses to moral dilemmas were less frequent than in males. However, similarly to the present work, the discrepancies on sex differences depending on the assessment instruments also suggest that self-reports are likely to promote participants to assume gender-role stereotypes and the consequent gender biases. This should be taken into account when interpreting the outcomes concerning sex differences and gender roles.

Another finding was that most of the students were not conscious of ageism; however, the feeling of guilt was higher in the questionnaire of older people. In a significant number of participants, the two open metacognition questions elicited a thought or even an action that may help raise awareness, take responsibility (students blame more themselves than society), and tackle ageism.

It has been observed that negative stereotypes are associated with low-quality care for older people. For this reason, different solutions had been suggested on an institutional level. These solutions include reducing stereotypes of older people in media, promoting activities that could unite different generations, and the most important: increasing students’ knowledge of aging through students’ education. In the last aspect, the participants did not yet receive such education and training, since the topic on geriatrics belongs to the most advanced courses of the medical degree, but they may be more predisposed to have better ageism stereotypes. In the case of the students of biology, aging is a topic scarcely adressed in most subjects where the content could make it feasible to do so. However, in most cases, the training courses about older people are based on the biomedical model and scarcely include psychological and social issues [47]. Moreover, other studies noticed that gerontology education would have to be available not only for medical students. It has to expand its offering to other areas such as engineering and business, as their future work will also impact the older population [48]. This was also the vision of some Spanish universities when they included ‘Healthy aging’ as a free-election topic in three different interuniversity programs, namely, Intercampus (with eight Catalonian universities of Xarxa Vicens Vives), Metacampus (with UOC, Universitat Oberta de Catalunya, an online university) and Campus Global (with UPM, Universidad Politécnica de Madrid, the oldest and largest of all Technical Universities in Spain, with students of architecture and telecommunications). Additionally, the topic is part of Universitat a l’Abast, a specific senior life-long educational program at our university, similar to other educational gerontology initiatives and European projects (SIforAGE and TOY) successfully implemented in other universities [49].

Most intergenerational programs have been designed as interventions to promote active aging and their outcomes compile the experiences and perspectives of older adults [50], showing effectiveness in improving the mental health of this population [51], regardless of the intervention mode employed [52]. In the present work, the focus was on the young people, as a direct approach to the actors. We used the intergenerational relationships as a tool to unveil ageism toward ‘the other’ older people with whom they do not have family/affective bonds, at a sensitive madurative age since they are in an educational program to build their future professional roles. Similarly, other direct strategies aimed at exerting such an effect in the undergraduate population include creative story telling [53,54].

With regard to limitations, most can be related to methodological issues potentially related to selection, measurement and analysis bias. Thus, the first to discuss are limitations intrinsic to convenience samples, the biomedical scenario of the participants and the common feminization of these degrees. Taking into account that Spain is a familyist country, the relevance of such cultural aspects could also be considered to positively influence the results and be a limitation for crosscultural translation. As reported, no differences between the stereotypes recorded in the universities were found. Other limitations include that the present retrospective analysis on metacognition is dependent on the previous survey and its temporal frames. Still, results are in agreement with other studies in the field. Additionally, as mentioned before, the voluntary-response sampling may elicit bias in the self-reports, mainly those related to gender roles. Here, one could also discuss whether the two open questions investigate metacognition or self-reflection. In fact, reflection is defined as ‘an act of looking back in order to process experiences and metacognition, a particular form of reflection consisting of ‘thinking about one’s thinking’ in order to grow [55]. Both are used to improve student learning [56] and other studies on racism also show that engaging students in metacognitive and meta-affective reflections enhances learning in race-related courses [57]. Similarly, the use of fictional characters to examine social attitudes toward aging and older people has recently been used to challenge ageist assumptions and negative stereotyping [58].

On the other hand, it has been reported that the positive attitudes of faculty members may also influence the positive perception of older people that their fellows have [34,59]. In the present work, this aspect was minimized since the survey was administered by a member of another university together with the university host.

In our opinion, further studies are needed to determine the impact of bio-psycho-social education on metacognition, stereotypes of older people as a way to tackle ageism in the exceptional scenario of 2020–2030 as the Decade of Healthy Aging. Nevertheless, the first UN Global Report on Ageism [3] has defined three critical dimensions of ageism, namely, cognitive (stereotypes), emotional (prejudice), and attitudes (discrimination) toward others, and pointed at educational activities and intergenerational interventions, together with policies, laws, and other approaches as the best strategies to tackle and combat it.

## Figures and Tables

**Table 1:** Students’ answers in the ten different items with regards to the older people and the grandparents’ questionnaires.

Existence of Metacognition					
**Questionnaire**	% No	% Yes			
Older people	6.7	93.3			
Grandparents (***)	15.9	84.1			
What feelings had answering the questionnaires produced?					
**Questionnaire**	Very negative	Negative	Neutral	Positive	Very positive
Older people	13.5	6.6	40.8	37.8	1.3
Grandparents (**)	6.2	24.5	17.5	42.7	9.1
Who is the subject of the thought?					
**Questionnaire**	No one	I	We	Older people	
Older people	2.3	97	0.7	none	
Grandparents (n.s)	2.9	96.7	none	0.4	
What role appeared in the thought?					
**Questionnaire**	No role	I-grandparents	We-grandparents	I-older people	We-older people
Older people	62.5	17.4	0.3	11.5	8.2
Grandparents (***)	56.2	40.9	none	0.7	2.2
Is there ageism awareness? Who is guilty of this ageism?					
**Questionnaire**	No	No guilty	I	We-society	Older people
Older people	83.9	2.0	7.2	6.6	0.3
Grandparents (***)	91.6	0.7	4.7	2.9	none
Is the feeling translated into a thought or an action?					
**Questionnaire**	Nothing	Thought	Action		
Older people	52.6	45.4	2.0		
Grandparents (n.s)	52.9	44.5	2.6		
What kind of older person is reflected in the answer?					
**Questionnaire**	None	Familiar	Social		
Older people	61.5	17.4	21.1		
Grandparents (**)	57.7	38.3	4.0		
What age is reflected in the answer?					
**Questionnaire**	None	+65 years	+80 years		
Older people	61.5	36.9	1.6		
Grandparents (n.s)	57.7	30.6	11.7		
Could answering the questionnaires help to tackle ageism?					
**Questionnaire**/	Neutral	Tackle ageism			
Older people	36.5	63.5			
Grandparents (***)	24.1	75.9			

Results are expressed as a percentage. Statistics: Marginal homogeneity test, *** *p* < 0.001; ** *p* < 0.01, vs. Older people; n.s., no statistically significant, *p* > 0.05.

## Data Availability

Available upon request. The original contributions presented in the study are included in the article, further inquiries can be directed to the corresponding author/s.
